# Thermodynamic and Interfacial Characterization of Petroleum Industry Surfactants: A Study on Critical Micelle Concentration and Interfacial Tension Behavior under Mild Conditions

**DOI:** 10.1002/open.202500066

**Published:** 2025-05-09

**Authors:** Rebeka Bejczi, Roland Nagy

**Affiliations:** ^1^ Department of MOL Hydrocarbon and Coal Processing University of Pannonia Veszprém H‐8200 Hungary

**Keywords:** colloidal study, critical micelle concentration, interfacial tension, surfactants, thermodynamics

## Abstract

Surfactants play a vital role in oil and gas applications, particularly in enhanced oil recovery (EOR), where interfacial tension (IFT) reduction and micellization are key to improving fluid mobility. This study aims to evaluate the interfacial and thermodynamic properties of five widely used petroleum surfactants to better understand their efficiency under mild reservoir‐like conditions. IFT is measured using the spinning drop tensiometer across a temperature range of 25–40 °C, while critical micelle concentrations (CMC) are determined via conductometric methods. Thermodynamic parameters of micellization are calculated from temperature‐dependent CMC data. The results show that micellization is an entropy‐driven process for all tested surfactants. The studied surfactants significantly reduce IFT values within the tested temperature range, underscoring their potential for practical application in EOR formulations. The study also confirms that accurate determination of CMC is essential in optimizing surfactant efficiency under varying environmental conditions.

## Graphical abstract

The interfacial and thermodynamic properties of five petroleum industry surfactants are evaluated under mild conditions. Interfacial tension is measured using spinning drop tensiometry, and critical micelle concentrations are determined conductometrically. The results demonstrate that micellization is entropy‐driven and confirm the efficiency of surfactants in reducing interfacial tension, supporting their application in enhanced oil recovery.

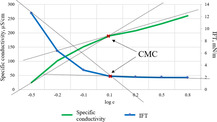

## 1 Introduction

The efficient recovery of hydrocarbons from increasingly complex geological formations presents significant challenges that necessitate the use of advanced chemical technologies. Among these, surfactants have emerged as indispensable agents in the petroleum industry, particularly in enhanced oil recovery (EOR), drilling fluids, and well‐stimulation treatments. Their multifunctional properties such as reducing interfacial tension (IFT), altering rock wettability, and stabilizing emulsions contribute directly to improved fluid mobility and oil displacement efficiency under reservoir conditions, which are often characterized by high salinity, elevated temperature, and pressure variations.^[^ ^1^, ^2^ ^]^

In recent years, surfactant research has shifted toward developing tailored molecular structures capable of withstanding these harsh environments while balancing economic viability and environmental sustainability. Accurate measurement of interfacial properties, especially IFT and critical micelle concentration (CMC), has become essential in designing surfactant systems optimized for field applications. Techniques such as the spinning drop method for IFT and conductometric analysis for CMC have proven to be reliable and precise tools for characterizing the thermodynamic behavior of surfactants under laboratory conditions that simulate those in oil fields.^[^ ^1^, ^2^ ^]^

The importance of surfactants in EOR is well established. They are known to significantly reduce the IFT between oil and water phases, a key factor in enhancing oil mobilization from porous rock structures. Several studies have shown that the efficacy of surfactants in this regard is highly dependent on their molecular composition and concentration, as well as on the specific reservoir conditions.^[^ ^1^, ^2^ ^]^ For example, lignin‐derived anionic–nonionic surfactants have been shown to maintain favorable interfacial properties across a range of salinity and temperature conditions,^[^ ^2^ ^]^ underlining the importance of tailored surfactant formulations.

In addition to IFT reduction, surfactants influence rock wettability another crucial factor in oil displacement. Their amphiphilic nature allows them to modify the surface energy of reservoir rocks, facilitating a transition from oil‐wet to water‐wet conditions, which enhances oil recovery.^[^ ^3^, ^4^ ^]^ Advances in methodology, such as contact angle measurements on carbonate rock surfaces, have provided deeper insights into the mechanisms by which surfactants act in realistic porous media.^[^ ^5^ ^]^

Innovation has also led to the development of advanced surfactant types, such as Gemini surfactants, which exhibit superior emulsification and thermal stability. These compounds have demonstrated strong potential in both laboratory and field settings, where stable emulsions are necessary for effective oil‐water separation, particularly under electrostatic treatment conditions.^[^ ^6^, ^7^ ^]^ Their synthesis, in parallel with the study of traditional surfactants, broadens the applicability of surfactant technology across diverse reservoir environments.^[^ ^8^ ^]^

Moreover, the integration of surfactants into multiphase flow systems has been investigated, with recent studies identifying formulations that reduce capillary pressure and improve fluid transport in mature reservoirs.^[^ ^9^ ^]^ Emerging trends also include the synergistic use of nanoparticles and surfactants to overcome adsorption losses and further enhance oil recovery.^[^ ^10^, ^11^ ^]^

Recent progress in surfactant science highlights their crucial importance in petroleum recovery processes. A thorough understanding of their interfacial and thermodynamic behavior under realistic reservoir conditions is vital for the design of efficient and dependable EOR systems. The aim of this study is to support the development of more effective surfactant‐based EOR systems by providing a detailed characterization of both the interfacial and thermodynamic properties of five industrially relevant surfactants. IFT measurements were carried out using the spinning drop method, while critical micelle concentrations were determined conductometrically over a temperature range of 25–40 °C. Based on these data, key thermodynamic parameters of micellization including standard Gibbs free energy, enthalpy, and entropy were calculated to better understand the temperature‐dependent behavior and entropy‐driven nature of micelle formation under reservoir‐like conditions. The findings contribute to the rational selection and optimization of surfactant formulations for EOR applications.

## 2 Theoretical Background

Surface tension and surface energy describe the same physical concept: molecules at a surface have fewer neighbors than those in the bulk, making their position less favorable. To minimize this, materials adopt shapes like spheres to reduce surface area. Surface tension (*γ*) is the work required to create a new unit of surface area (Equation (1))

(1) $$W_{\text{min}}=\gamma \Delta A$$

Surfactants reduce this work by adsorbing onto surfaces. Hydrophobic effects lead surfactants to migrate to the surface, where they form a monomolecular layer. The CMC is the point at which micelles start forming, with the system containing monomolecular layers, free molecules, and micelles in equilibrium. Below CMC, adsorption is dynamic, with continuous exchange between surface‐bound and free molecules. Surface tension relaxes to an equilibrium value over time, requiring surfactants to migrate, adsorb, and orient themselves.

Surface excess concentration, the ratio of surface particles to bulk particles, can be described thermodynamically (Equation (2) and (3))

(2) $$U=U^{\alpha }+U^{\beta }+U^{\sigma }$$

where *U* is the internal energy, *α* and *β* are the two phases, and *σ* is the interface.

The interfacial energy can be calculated as follows (Equation (3))

(3) $$U^{\sigma }=TS^{\sigma }+\gamma A+\sum \mu_{i}n_{i}^{\sigma }$$

where *T* is the temperature, *S* is the entropy, *γ* is the IFT, *A* is the surface area, μ is the chemical potential, and *n* is the number of moles.

At constant temperature, the relationship between IFT and surface excess concentration can be described by the following Equation (4)

(4) $$d\gamma =-\sum \Gamma_{i}^{\sigma }d\mu_{i}$$

Nonionic surfactants can also have their IFT defined as follows (Equation (5))

(5) $$d\gamma =-\Gamma^{\sigma }\text{RT dlna}$$

where *Γ* ^σ^ is the surface excess concentration of the solute at the interface, *R* is the universal gas constant, *T* is the temperature, and *a* is the activity of the solute in the solution.

For substances that strongly adsorb onto surfaces (e.g., surfactants), a significant reduction in IFT can occur even without a substantial change in the bulk phase concentration. The practical utility of this relationship is that the relative surface adsorption, or surface activity, of a surfactant can be determined by measuring the IFT.^[^ ^12^ ^]^

**Figure 1:**
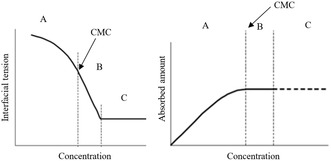
The change in IFT and the amount adsorbed as a function of concentration.^[^ ^12^ ^]^

**Figure** **1** illustrates the decrease in IFT as a function of surfactant concentration and demonstrates how the Gibbs equation can be used to quantitatively describe surface adsorption.

At low concentrations, the surface tension continuously decreases with the increase in adsorbed surfactant molecules (A). Upon reaching the CMC (boundary AB), the change in tension becomes nearly linear (B). Subsequently, it reaches a minimum value and does not decrease further with additional increases in concentration. To directly measure the surface concentration, a combination of tensiometry and neutron reflectometry techniques is used.^[^ ^13^ ^]^

The Gibbs model is widely used for liquid–liquid and gas–liquid systems, but other theories like Langmuir, Szyszkowski, and Frumkin are also important. It simplifies micelle formation by treating the interface as a separate phase. Above the CMC, the number of free monomers is stable due to complex interactions. The Gibbs–Helmholtz equation describes micelle formation, characterized by a small positive enthalpy change (ΔHm), a large positive entropy change (ΔSm), and a negative free energy change (ΔGm) at room temperature.^[^ ^14^ ^]^

(6) $$\Delta G_{m}=\Delta H_{m}-T\Delta S_{m}$$

Micelle formation occurs through a series of sequential equilibrium processes. The micelle formation equilibrium constant (*K*_m_) can be defined by the following Equation (7)

(7) $$K_{m}=\frac{\left[\text{micelles}\right]}{{\left[\text{monomers}\right]}^{n}}=\frac{\left[S_{n}\right]}{{\left[S\right]}^{n}}$$

where *n* is the aggregation number, that is, the number of monomers in the micelles, and *S* is the molar concentration of the surfactant.

The micelle formation free energy can be expressed in terms of the equilibrium constant, and by rearranging the equation, other thermodynamic parameters such as ΔHm and ΔSm can also be determined. For large aggregation numbers (*n* ≈ 100), calculations using the critical micelle concentration provide a good approximation (Equation 8).^[^ ^15^ ^]^

(8) $$\Delta G_{m}=-\text{RT ln}K_{m}≅\text{RTln xcmc}$$

Studies of the literature reveal a significant relationship between surface tension and the energetic properties of surfactants, which can provide important information about the structure of the surfactants.

The aim of this work is to investigate the colloidal chemistry of surfactants and surfactant mixtures. Specifically, the study focuses on examining the surface tension of aqueous solutions of the surfactants and surfactant mixtures under investigation. Based on the obtained surface tension measurements, the research seeks to estimate the associated energy functions. The presented method may be used to determine the energetic characteristics of substances that cannot be found in the literature (e.g., new compounds, mixtures, etc.).

## 3 Experimental Section

Commercially available surfactants were used for the experiments. The properties of the utilized surfactants are depicted in **Table** **1**.

**Table 1:** Main properties of the surfactants used.

Surfactant	S‐1	S‐2	S‐3	S‐4	S‐5
Manufacturer	Kao Chemicals	Merck	Merck	Ronas Chemicals	Clariant
Country	Germany	Germany	Germany	China	Switzerland
Type	anionic	anionic	anionic	nonionic	nonionic
Type of active matter	Sodium lauryl ether sulfate properties	Lauryl sulfate sodium salt	Sodium dodecylbenzene sulfonate	Cocamide diethanolamide	Fatty alcohol polyglycol ether
Phase	Liquid	Liquid	Liquid	Liquid	Liquid
Active matter concentration, %	68–72	67–70	75–80	80–85	100
pH	7–9	9	7–10.5	5–7	5–7.5

A series of solutions were prepared from each surfactant used and analyzed at temperatures ranging from 25 to 40 °C.

Crude oil from the South Hungary oil field was used in the measurements. The key properties of this crude oil are summarized in **Table** **2**.

**Table 2:** The most important properties of the crude oil used for the tests.

Property	Value
Density, g cm^−3^ [*d*_254_]	0.8242
API density	38.7
Dynamic viscosity, mPas [25 °C]	45.0
Kinematic viscosity, mm^2^ s^−1^ [25 °C]	51.5
Kw [Watson]	12.8
Characteristic [based on Kw]	Paraffinic
Approx. mean average boiling point [°C]	410

Based on the values presented in the table, the crude oil used for the tests was considered to be a light, paraffinic crude oil.

## 4 Methods

The surface chemical properties of aqueous surfactant solutions have been extensively studied, both due to scientific interest and practical importance.^[^ ^16^, ^17^, ^18^ ^]^ Surfactants derived from unsaturated fatty acids, particularly oleic acid, are among the most popular. The behavior of aqueous solutions of surfactants with varying carbon chain lengths has been investigated across a wide range of concentrations and temperatures, which is especially relevant for practical applications. This work involved examining different types of surfactants through surface tension measurements and aimed to determine how their structure influences other properties.

### 4.1 Determination of Interfacial Tension

The IFT between the aging crude oil and aqueous surfactant solutions was measured using a Spinning Drop Tensiometer (SDT) from Krüss (Germany). The surfactant solution was added to a glass tube, and then an oil droplet was injected into the center of the water phase. The experiment was conducted at temperatures ranging from 25 to 40 °C. In this method, the shape of the drop within the spinning system is influenced by the applied known forces. The IFT can be determined from the drop geometry, angular velocity, and the properties of the two phases (oil phase and aqueous phase). Prior to conducting the measurements, the SDT was calibrated using standard reference liquids to ensure accuracy and precision. The spinning drop method was chosen because it is widely used for the selection of petroleum surfactants and their mixtures.

### 4.2 Determination of Specific Conductivity

Conductivity measurements were performed using the Denver Instrument Model 250 device from Sartorius Group (Germany). This instrument is capable of measuring conductivity, salinity, resistance, and TDS. It automatically calibrates the measured conductivity values using predefined cell constants and selects the appropriate units accordingly. Prior to use, the instrument was calibrated with standard conductivity solutions according to the manufacturer's instructions to ensure accuracy and reproducibility. The device is applicable within the measurement range of 0 to 3 × 10^5^ S cm^−1^.

## 5 Results

Following the determination of surface tension and electrical conductivity, the CMC and surface adsorption were assessed. Subsequently, the thermodynamics of micelle formation were analyzed and interpreted based on these measurements.”

### 5.1 Interfacial Tension

**Figure 2:**
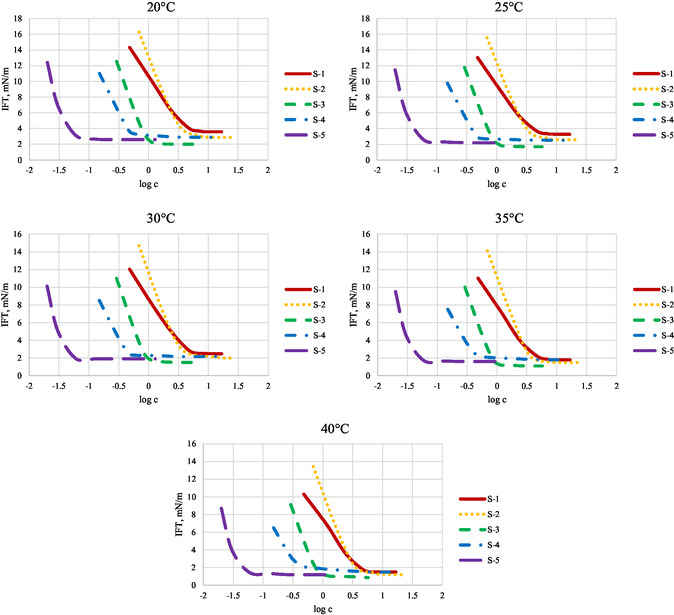
IFT values at different temperatures.

**Figure** **2** summarizes the IFT values for surfactant solutions measured at temperatures ranging from 20 to 40 °C.

It is evident that for all surfactants, the IFT decreases with increasing temperature. The highest IFT value recorded was for S‐1 at 20 °C, with a measurement of 16.254 mN m^−1^. Conversely, the lowest IFT value was observed for S‐3 at 40 °C, at 0.865 mN m^−1^.

The IFT values are plotted against the logarithmic concentration values, clearly showing the inflection point at the CMC. For convenience in further calculations, the concentrations are reported in mmol/L instead of g/L.

### 5.2 Specific Conductivity

**Figure 3:**
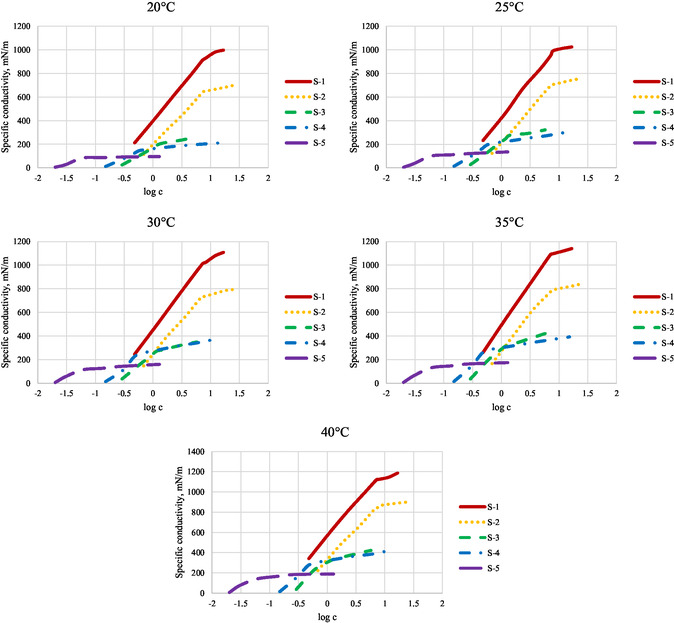
Specific conductivity at different temperatures °C.

To determine the CMC, specific conductivity measurements were also conducted. The values measured in the solutions are summarized in the following **Figure** **3**.

The specific conductivity values are plotted against the logarithmic concentration values, similar to the method used for IFT, clearly showing the inflection point at the CMC, with concentrations reported in mmol/L for convenience in further calculations.

### 5.3 Determination of Critical Micelle Formation Concentration

The CMC is essential for surfactant applications, as surface tension levels off above this point, indicating the optimal concentration for various processes. To account for losses such as adsorption, surfactants are often used at concentrations above the CMC, typically ranging from 0.5 to 5% by mass, depending on the desired effect.

**Figure 4:**
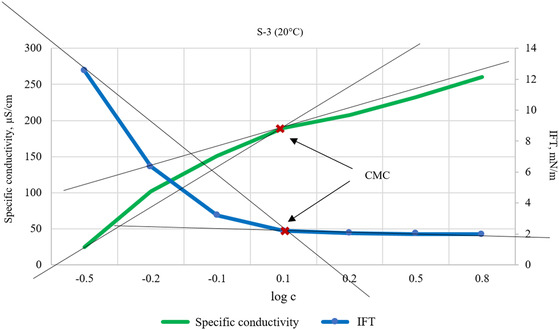
Determination of CMC value.

Surface adsorption characteristics, such as surface excess concentration and molecular area, can be derived from the CMC. To determine the CMC, properties are plotted against the logarithm of surfactant concentration. Surface tension initially decreases linearly with increasing concentration but stabilizes after the CMC, while conductivity increases continuously with concentration, with a reduced rate of increase past the CMC. The intersection of the linear regions in these plots identifies the CMC (**Figure** **4**).

As the concentration increases, the rate of decrease in IFT slows down, while ionic dissociation continues. These observations indicate a significant association. Micelle formation and adsorption are the two key phenomena that significantly reduce the interfacial energy of surfactant solutions. To accurately determine the CMC, two different types of measurements were performed. If one method does not provide a clear result, the other method helps in establishing it. The method for determining the CMC is illustrated in the figure (Figure 4). The CMC values measured using different methods are summarized in **Table** **3**.

**Table 3:** CMC values measured using different methods.

Surfactant	Temperature [°C]	CMC by IFT [g L^−1^]	CMC by specific conductivity [g L^−1^]	Deviation [g L^−1^]
S‐1	20	3.6	3.59	0.01
	25	3.38	3.38	0
	30	3.2	3.2	0
	35	2.9	2.79	0.11
	40	3.07	2.96	0.11
S‐2	20	2.22	2.23	0.01
	25	2.2	2	0.2
	30	2	2	0
	35	1.8	1.77	0.03
	40	2.01	2.09	0.08
S‐3	20	0.44	0.46	0.02
	25	0.4	0.44	0.04
	30	0.42	0.39	0.03
	35	0.39	0.39	0
	40	0.41	0.4	0.01
S‐4	20	0.23	0.22	0.01
	25	0.19	0.19	0
	30	0.18	0.16	0.02
	35	0.17	0.17	0
	40	0.16	0.17	0.01
S‐5	20	0.02	0.027	0.007
	25	0.021	0.022	0.001
	30	0.02	0.02	0
	35	0.017	0.021	0.004
	40	0.018	0.018	0

Table 3 shows that the differences in CMC at various temperatures are small, with identical values often observed.

For further calculations, the arithmetic mean of the values obtained from the different methods was used.

**Figure 5:**
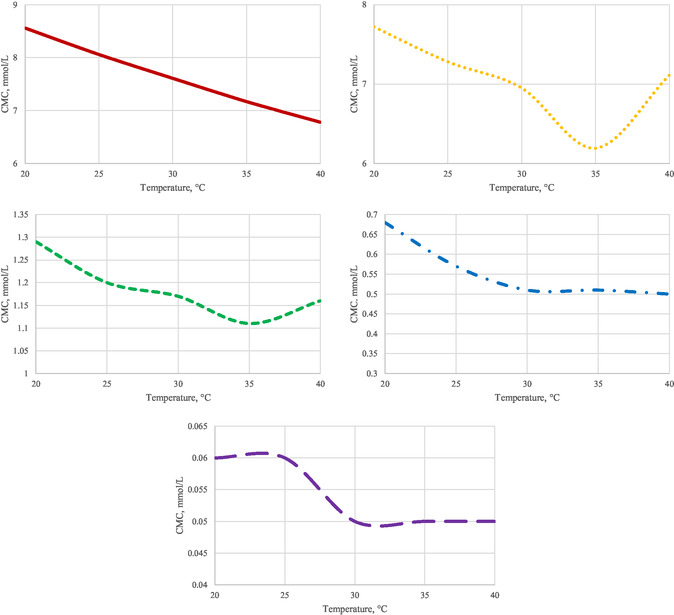
Micellization optimum curves of surfactants.

**Figure** **5** depicts the temperature dependence of CMC for the surfactants. Typically, the CMC‐temperature curve reveals a minimum.

At first, the CMC decreases as the temperature rises, due to a decrease in the molecules’ hydrophilicity, which promotes micelle formation. However, at higher temperatures, the structured water molecules surrounding the hydrocarbon chains are displaced, reducing the hydration of the hydrophobic regions.

This reduction in hydration is detrimental to micelle formation, causing the CMC to increase again at elevated temperatures. Thus, temperature initially decreases hydrophobic effects but eventually worsens hydrophilic properties.^[^ ^19^ ^]^

At low surfactant concentrations, the adsorption of surfactants on a solid surface is governed by the charge of the electrical double layer of the surface. Electrostatic effects arise between the head groups of the surfactant and the charge of the solid surface. At low concentrations, surfactant molecules adsorb as monomers onto the rock surface.

As the surfactant concentration increases, these monomers begin to aggregate and associate to form micelles. In this case, hydrophobic effects influence the adsorption process. The CMC represents the maximum concentration of monomers and the free energy of micelle formation. The lower the CMC, the more stable the micelle, and the slower the molecules incorporate into or depart from the micelle. The results indicate that Tmin (temperature where CMC is the lowest) increases with the hydrophilicity of the surfactant molecules, while it decreases with an increase in the length of the hydrocarbon chain, that is, the hydrophobic properties. Thermal motion enhances molecular movement, thus facilitating the formation of micelles.^[^ ^20^ ^]^

### 5.4 Estimation of Surface Adsorption

Surfactant adsorption onto solid matrices, such as rock or clay, can reduce surfactant concentrations and diminish the efficiency of industrial processes like EOR, where surfactants lower oil‐water IFT to enhance oil recovery. Retention of surfactants on solid surfaces results in losses and reduced efficiency, affecting the cost‐effectiveness of chemical treatments.

Surfactant adsorption depends on the surface charge of the solid material, with anionic surfactants commonly used to minimize adsorption on negatively charged surfaces. Factors such as pH, temperature, ionic strength, and electrolyte concentration all influence adsorption. At lower concentrations, surfactants adsorb as monomers, while higher concentrations promote micelle formation. The specific type of surfactant and the characteristics of the solid matrix significantly impact adsorption behavior.^[^ ^21^, ^22^, ^23^, ^24^, ^25^, ^26^, ^27^ ^]^

(9) $$\Gamma_{\text{max}}=\frac{1}{\text{RT}}\left(\frac{\text{dλ}}{\text{d lnc}}\right)$$

where $\Gamma_{max}$ is the maximum concentration of the solute at the interface [mol m^−2^], *R* is the universal gas constant (8,314 J molK^−1^), *T* is the temperature [K], $\left(\frac{\text{dλ}}{\text{d lnc}}\right)$ is the slope of the isotherm, λ is the IFT [mN/m], and *c* is the surfactant concentration in the solution [mol/m^3^].

This formula helps estimate adsorption properties from IFT/concentration data. Decreasing IFT with increasing concentration indicates higher adsorption, while negative adsorption suggests solute depletion at the interface.^[^ ^28^, ^29^ ^]^

**Table** **4** compares the IFT values calculated from the measurements with those found in the literature.

**Table 4:** Surface adsorption values at different temperatures.

*T* [°C]	*Γ* [mol cm^−2^ × 10^10^]	S‐1	S‐2	S‐3	S‐4	S‐5
20	Calculated	2.03	3.78	3.67	3.72	3.08
	Literature	1.82	3.47	3.59	–	–
	Deviation	0.21	0.01	0.08	–	–
25	Calculated	1.9	3.37	3.49	2.55	2.93
	Literature	1.68	3.41	3.51	–	–
	Deviation	0.22	−0.04	−0.02	–	–
30	Calculated	1.8	3.26	3.32	2.27	2.77
	Literature	1.63	3.35	3.45	–	–
	Deviation	0.17	−0.09	−0.13	–	–
35	Calculated	1.66	3.09	3.12	2.05	2.68
	Literature	1.6	3.15	3.26	–	–
	Deviation	0.06	−0.06	−0.14	–	–
40	Calculated	1.49	2.89	3.01	1.87	2.55
	Literature	1.54	3	3.03	–	–
	Deviation	−0.05	−0.11	−0.02	–	–

**Figure 6:**
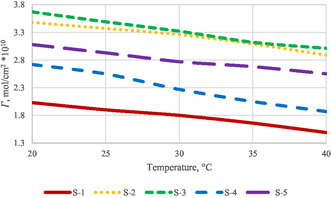
Adsorbed quantities as a function of temperature.

From the table, it is evident that for all surfactants, the amount adsorbed on the surface decreases with increasing temperature. The strong, directed adsorption of surfactants in a monomolecular layer at the interface is termed surface activity. Surface activity is a dynamic phenomenon regulated by adsorption and thermal motion. The temperature dependence of the adsorbed quantities is illustrated in **Figure** **6**.

The graph clearly shows that the number of adsorbed molecules decreases with increasing temperature. This occurs because adsorption is always exothermic and releases heat. Adsorption equilibrium is established during the contact between the adsorbent and the adsorptive, and it is dynamic in nature. Therefore, particles bound to the surface detach due to thermal motion, and new particles take their place. As the temperature increases, thermal motion intensifies, favoring the desorption process. Surface adsorption can also be characterized by the surface area occupied by surfactant particles. The following equation establishes the relationship between the maximum amount that can be adsorbed on the surface and the area occupied by the molecules.^[^ ^30^ ^]^

(10) $$A_{\text{min}}=\frac{1}{\Gamma_{\text{max}}N_{A}}$$

where $\Gamma_{max}$ is the maximum concentration of the dissolved substance at the interface [mol/m^2^], $A_{\text{min}}$ is the molecular area [m^2^ db^−1^], and *N*_A_ is the Avogadro's number (6 × 10^23^ db mol^−1^).

The equation shows that as the quantity of molecules adsorbed at the interface increases, the surface area occupied by a single molecule decreases. The calculated surface area values are summarized in **Table** **5**.

**Table 5:** Calculated surface area values.

	A_min_ [A° db^−1^]				
	Temperature [°C]				
Surfactant	20	25	30	35	40
S‐1	82.01	87.77	92.37	100.25	112.01
S‐2	47.93	49.45	51.2	53.94	57.67
S‐3	45.4	47.69	50.16	53.42	55.46
S‐4	61.3	65.44	73.31	81.16	88.94
S‐5	54.18	56.86	60.11	62.26	65.37

The largest surface area is occupied by the S‐1 particles, which have the highest molecular weight, at 40 °C, while the smallest surface area is associated with the S‐3 particles at 20 °C.

### 5.5 Thermodynamics of Micelle Formation

The standard free energy of adsorption for surfactants is the energy required for a surfactant molecule to move from the bulk phase of the solution to the infinitely diluted adsorption layer. For nonionic surfactants, the free energy of micellization, ΔGm, can be described by the following equation

(11) $$\Delta G_{m}=-\text{RT lnx CMC}$$

where Δ*G*_m_ is the free energy change of micelle formation, and × CMC is the mole fraction of the surfactant at the critical micelle concentration.

**Figure 7:**
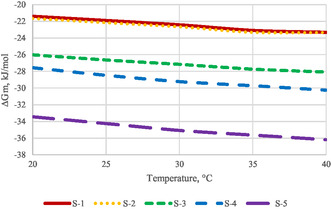
Temperature dependence of free energy of micellization.

The change in free energy of micellization represents the energy change that occurs when the free molecules of a surfactant in an aqueous solution aggregate to form micelles. The relationship between the calculated free energy values and temperature is illustrated in **Figure** **7**.

The micellization entropy can be estimated from the temperature dependence of the free energy of micellization using the following thermodynamic relationship.

(12) $$\Delta S_{m}=-\left(\frac{\partial \Delta G_{m}}{\partial T}\right)$$

Using this, the enthalpy of micellization can also be calculated using the following equation

(13) $$\Delta G_{m}=\Delta H_{m}-T\Delta S_{m}\;$$

The thermodynamic parameters of micellization are summarized in **Table** **6**.

**Table 6:** Thermodynamic parameters of micellization.

Surfactant	Temperature [°C]	*X*_CMC_*10^5^	*Δ*Gm [kJ mol^−1^]	*Δ* Hm [KJ mol^−1^]	T*Δ*Sm [kJ mol^−1^]	*Δ*Sm [kJ molK^−1^]
S‐1	20	15.4	−21.4	8.58	29.97	0.1
	25	14.5	−21.9	8.19	30.1	
	30	13.7	−22.42	7.88	30.3	
	35	12.9	−23.09	7.28	30.37	
	40	12.2	−23.32	–	–	
S‐2	20	13.9	−21.65	7.36	29.01	0.1
	25	13.1	−22.16	6.95	29.11	
	30	12.5	−22.65	6.59	29.24	
	35	11.15	−23.32	5.94	29.26	
	40	12.8	−23.34	–	–	
S‐3	20	2.32	−26.01	10.5	36.36	
	25	2.16	−26.63	10.05	36.68	0.12
	30	2.1	−27.15	9.85	37	
	35	1.99	−27.73	9.54	37.27	
	40	2.08	‐28.07	–	–	
S‐4	20	1.23	−27.56	11.85	39.41	0.13
	25	1.03	−28.47	10.99	39.46	
	30	0.92	−29.23	10.8	40.03	
	35	0.92	−29.72	10.32	40.04	
	40	0.9	−30.25	–	–	
S‐5	20	0.11	−33.44	8.34	41.78	0.14
	25	0.1	−34.25	7.89	42.14	
	30	0.09	−35.08	7.22	42.3	
	35	0.09	−35.64	6.81	42.44	
	40	0.09	−36.19	–	–	

The table shows that free energy decreases with higher temperatures and lower CMC. The highest free energy is for surfactant S‐1 (−21.4 kJ mol^−1^), while the most negative value is for S‐5 at 40 °C (−36.19 kJ mol^−1^).

These thermodynamic parameters not only quantify surfactants but also reveal insights into the adsorption process. Data indicate that for both ionic and nonionic surfactants, TΔSm» ΔHm, suggesting that adsorption is driven more by entropy increase than by energy changes. This is due to the rearrangement of water molecules around hydrocarbon chains, which decreases system entropy. Micellization significantly increases entropy by creating a new phase within the bulk phase.

The spontaneous nature of micellization is evident from the thermodynamic parameters. As temperature rises, free energy increases, leading to larger micelles due to enhanced thermal motion and increased molecular spacing.

**Table** **7** compares these findings with literature values, and energy changes related to micellization were analyzed using Frumkin, van der Waals, and Helfand–Frisch–Lebowitz models.^[^ ^31^, ^32^, ^33^, ^34^, ^35^ ^]^

**Table 7:** Comparison of free energy values.^[^ ^29^, ^36^, ^37^, ^38^ ^]^

*T* [°C]	−*Δ*Gm [kJ mol^−1^]	S‐1	S‐2	S‐3	S‐4	S‐5
20	Calculated	21.4	21.65	26.01	33.44	27.56
	Literature	21.32	17.52	24.2	–	–
	Deviation	0.08	4.13	1.81	–	–
25	Calculated	21.91	22.16	26.63	34.25	28.47
	Literature	21.78	17.61	24.4	–	–
	Deviation	0.13	4.55	2.23	–	–
30	Calculated	22.42	22.65	27.15	35.08	29.23
	Literature	22.51	17.84	24.8	–	–
	Deviation	−0.09	4.81	2.35	–	–
35	Calculated	23.09	23.32	27.73	35.64	29.72
	Literature	23.3	17.92	25.1	–	–
	Deviation	−0.21	5.4	2.63	–	–
40	Calculated	23.32	23.34	28.07	36.19	30.25
	Literature	23.5	18.2	25.4	–	–
	Deviation	−0.18	5.14	2.67	–	–

The free energy values from various thermodynamic models are closely approximated by a simplified model, with surfactant S‐1 showing the best match. The CMC and related thermodynamic properties offer insight into surfactant effectiveness. As surfactant concentration increases, IFT decreases as surfactant molecules adsorb onto surfaces, reducing repulsive interactions at the interface. Once the surface is fully covered, IFT stabilizes, and surfactant molecules in the bulk phase form micelles.

A surfactant's effectiveness is often characterized by its CMC, the concentration at which IFT drops significantly or micellization begins. Surfactants are typically used above the CMC to account for losses due to adsorption.

The micellization free energy, derived from the CMC and temperature, reflects how favorable micellization is and the stability of the micelles. A negative free energy indicates that micellization is spontaneous and exothermic, meaning it releases energy. Lower free energy values suggest that the micellar state is more stable and favorable due to increased disorder in the system.

## 6 Summary

This study investigated five surfactants SLES, SDS, SDBS, Genapol L3, and Coconut DEA across varying temperatures and concentrations, focusing on their IFT and CMC values. The IFT was measured using the spinning drop method, and CMC values were determined through conductance measurements at each temperature, offering insight into the surfactants’ efficiency.

The thermodynamic analysis revealed that micellization is primarily driven by an increase in entropy rather than an energy gain. This was reflected in the negative Gibbs free energy (ΔGm) values, indicating that micelle formation is a spontaneous process. The enthalpy (ΔHm) and entropy (ΔSm) changes further revealed that the adsorption process is exothermic, with a decrease in the number of adsorbed molecules as the temperature increased. Additionally, the data showed that at 40 °C, surfactant S‐1 exhibited the highest surface area per molecule, while S‐3 had the smallest surface area at 20 °C.

The study also highlighted the direct relationship between surfactant concentration and IFT, with the adsorption of surfactant molecules causing a decrease in IFT until micellization begins. The CMC value marks the concentration beyond which further reduction in IFT is not possible, and micelle formation becomes dominant in the bulk phase. This finding emphasizes that surfactant efficiency is largely determined by its CMC, and surfactant concentrations above the CMC are typically utilized in practical applications to account for losses due to adsorption.

In conclusion, the study provides essential thermodynamic data for understanding surfactant behavior, with spontaneous micellization and lower Gibbs free energy associated with more stable micellar structures. Future studies could extend these findings by investigating surfactant behavior at solid–liquid interfaces, providing a more comprehensive understanding of their performance in a variety of environments.

## Conflict of Interest

The authors declare no conflict of interest.

## Data Availability

The data that support the findings of this study are available from the corresponding author upon reasonable request.
